# Changes in the Electrically Evoked Compound Action Potential over time After Implantation and Subsequent Deafening in Guinea Pigs

**DOI:** 10.1007/s10162-022-00864-0

**Published:** 2022-08-10

**Authors:** Dyan Ramekers, Heval Benav, Sjaak F. L. Klis, Huib Versnel

**Affiliations:** 1 Department of Otorhinolaryngology and Head & Neck Surgery, University Medical Center Utrecht, Utrecht University, Room G.02.531, P.O. Box 85500, 3508 GA Utrecht, the Netherlands; 2 UMC Utrecht Brain Center, Utrecht University, Utrecht, the Netherlands; 3 MED-EL Elektromedizinische Geräte GmbH, Innsbruck, Austria

**Keywords:** Auditory nerve, Hearing loss, Neurodegeneration, eCAP, Cochlear implant, Cochlear health

## Abstract

The electrically evoked compound action potential (eCAP) is a direct measure of the responsiveness of the auditory nerve to electrical stimulation from a cochlear implant (CI). CIs offer a unique opportunity to study the auditory nerve’s electrophysiological behavior in individual human subjects over time. In order to understand exactly how the eCAP relates to the condition of the auditory nerve, it is crucial to compare changes in the eCAP over time in a controlled model of deafness-induced auditory nerve degeneration. In the present study, 10 normal-hearing young adult guinea pigs were implanted and deafened 4 weeks later, so that the effect of deafening could be monitored within-subject over time. Following implantation, but before deafening, most examined eCAP characteristics significantly changed, suggesting increasing excitation efficacy (e.g., higher maximum amplitude, lower threshold, shorter latency). Conversely, inter-phase gap (IPG) effects on these measures – within-subject difference measures that have been shown to correlate well with auditory nerve survival – did not vary for most eCAP characteristics. After deafening, we observed an initial increase in excitability (steeper slope of the eCAP amplitude growth function (AGF), lower threshold, shorter latency and peak width) which typically returned to normal-hearing levels within a week, after which a slower process, probably reflecting spiral ganglion cell loss, took place over the remaining 6 weeks (e.g., decrease in maximum amplitude, AGF slope, peak area, and IPG effect for AGF slope; increase in IPG effect for latency). Our results suggest that gradual changes in peak width and latency reflect the rate of neural degeneration, while peak area, maximum amplitude, and AGF slope reflect neural population size, which may be valuable for clinical diagnostics.

## Introduction

Auditory nerve degeneration following substantial hair cell loss is well documented for several species, including humans. In guinea pigs, the events following ototoxic trauma have been well documented: substantial hair cell loss results in immediate loss of acoustic hearing (Versnel et al. 2007) and is followed by gradual collapse of the organ of Corti (Tisi et al. 2022) and secondary degeneration of the spiral ganglion cells (SGCs) that form the auditory nerve within weeks (Ylikoski et al. 1974; Webster and Webster 1981; Versnel et al. 2007; van Loon et al. 2013; Ramekers et al. 2015a, 2020). Since cochlear implants (CIs) target the auditory nerve directly, its functional integrity is important for optimal hearing restoration with a CI. Therefore, in addition to exploring strategies that are aimed at the preservation of the auditory nerve (e.g., Miller et al. 1997; Wise et al. 2011; Pinyon et al. 2014; Ramekers et al. 2015a; Schwieger et al. 2020; Vink et al. 2020), the development of non-invasive methods for objective assessment of auditory-nerve integrity is essential.

In standard clinical settings, electrically evoked compound action potentials (eCAPs) can be recorded using the patient’s own CI, which uniquely provides a direct assessment of the electrophysiological status of the human auditory nerve (for a review see He et al. 2017). Hence, numerous clinical studies have revealed that the eCAP can be predictive of outcome measures for CI users. It has for instance been reported that larger eCAP amplitudes (e.g., DeVries et al. 2016; Scheperle 2017) and steeper slopes of the eCAP amplitude growth function (AGF) (e.g., Brown et al. 1990; Kim et al. 2010) are associated with better speech perception scores. In turn, animal studies have shown that the aforementioned eCAP measures, among others, correlate well with SGC survival after deafening (e.g., Ramekers et al. 2014; Pfingst et al. 2017; Schvartz-Leyzac et al. 2020a; Vink et al. 2020), thereby providing indirect evidence for the importance of numerical survival of SGCs for speech perception performance with a CI. More direct evidence is provided by human histopathological studies (e.g., Seyyedi et al. 2014; Kamakura and Nadol 2016) – showing that speech perception scores are positively correlated with SGC survival. This relationship, however, is not entirely undisputed, since there have also been reports of a negative correlation between the two (e.g., Nadol et al. 2001; Fayad and Linthicum 2006), as well as several studies that did not find a significant correlation (see Cheng and Svirsky (2021) for a meta-analysis).

Absolute eCAP measures such as the amplitude, slope, and threshold are believed to be affected by several non-neural factors, including intracochlear tissue growth, electrode impedance, electrode array design, and electrode-neuron distance (e.g., Pfingst et al. 2015; Schvartz-Leyzac et al 2020b). One approach to cancel out such confounding factors is to evaluate not the absolute eCAPs, but the difference in neural response to two or more distinct stimuli – while all non-neural factors are necessarily kept constant. Examples of such strategies are multi-pulse paradigms with which responses to odd- and even-numbered pulses can be compared (e.g., Carlyon and Deeks 2015; Ramekers et al. 2015a, b; Vink et al. 2020; Riggs et al. 2021), variation of stimulus polarity in order to assess polarity sensitivity (e.g., Rattay et al. 2001; Undurraga et al. 2010; Joshi et al. 2017; Hughes et al. 2018; Jahn and Arenberg 2019; Brochier et al. 2021), and variation of the inter-phase gap (IPG) in the biphasic current pulse (Prado-Guitierrez et al. 2006; Ramekers et al. 2014, 2015a; Schvartz-Leyzac and Pfingst 2016, 2018; Hughes et al. 2018; Schvartz-Leyzac et al. 2019, 2020a, b; He et al. 2020; Vink et al. 2020; Brochier et al. 2021; Imsiecke et al. 2021).

Varying the IPG creates a difference in eCAP measures, referred to as the “IPG effect” for these measures, of which the magnitude has been shown to correlate with SGC survival in deafened animals (Prado-Guitierrez et al. 2006; Ramekers et al. 2014, 2015a; Schvartz-Leyzac et al. 2019, 2020a; Vink et al. 2020). It has been shown that in humans, the IPG effects on eCAP threshold and slope of the eCAP AGF are less dependent (if at all) on the electrode-neural distance than the absolute eCAP threshold or slope (Schvartz-Leyzac et al. 2020b), which has confirmed that IPG measures are indeed better comparable across subjects than absolute eCAP measures.

In previous studies, we observed changes in both absolute eCAP measures and eCAP IPG effects after deafening, by recording eCAPs in animals that were either normal-hearing, or deafened 2, 6, or 14 weeks earlier (Ramekers et al. 2014, 2015a). Straightforward findings included a consistent decrease in eCAP amplitude and AGF slope after deafening, but more elusive findings included an initial increase in both threshold and latency after 2 weeks, but a marked decrease another 4 weeks later. IPG effects on these measures on the other hand appeared to consistently follow a linear course, either increasing (amplitude, latency, and dynamic range) or decreasing (slope and level_50%_, i.e., the current level required to evoke an eCAP with 50 % of the maximum amplitude) following SGC loss after ototoxic deafening. Schvartz-Leyzac et al. (2019) recorded eCAPs over time in chronically implanted normal-hearing and deafened guinea pigs, but did not have any data earlier than 2 weeks after deafening. Therefore, in the present study, we recorded eCAPs longitudinally after implantation in normal-hearing guinea pigs that were subsequently deafened. Both the initial effect of loss of hair cells and collapse of the organ of Corti as well as the delayed effect of SGC degeneration on the eCAP morphology, latency, and amplitude-derived measures could therefore be evaluated. By monitoring within-animal changes in eCAPs over time, we have been able to address the question whether changes in eCAP measures – including IPG effects – occur gradually in resemblance to the loss of SGCs, or whether these changes occur abruptly at specific critical time points in the degeneration process.

## Methods

### Animals and Experimental Design

Ten young adult female albino guinea pigs (Dunkin Hartley; Envigo, Horst, the Netherlands) were kept under standard housing conditions throughout the experiment (food and water ad libitum; lights on between 7:00 a.m. and 7:00 p.m.; temperature 21 °C; humidity 60 %). All experimental procedures were approved by the Dutch Central Authority for Scientific Procedures on Animals (CCD 11500201550 and 1150020174315). There were three reasons for using only female guinea pigs in this study, all of which acknowledged by the CCD. First, social housing is easier (i.e., no fighting); second, females are easier to handle (e.g., for awake recordings); third, females are less active/aggressive so that external parts of implants are less likely to come off or be tampered with.

All 10 animals were subjected to the same series of experimental procedures, starting with unilateral cochlear implantation after verification of normal hearing (day -28). Four weeks after implantation, the animals were systemically ototoxically deafened (day 0), and another 7 weeks later, they were terminated for histological analysis of their cochleas (day 49). eCAPs were recorded weekly during these 11 weeks (i.e., 12 weekly recording sessions). Three additional sessions were scheduled at days 1, 2, and 4 after deafening, bringing the total to fifteen identical eCAP recording sessions.

### Cochlear Implantation

The animals’ right ears were implanted with an intracochlear electrode array largely following a previously described procedure (Ramekers et al. 2015a). Animals were anesthetized by intramuscular injection of dexmedetomidine (Dexdomitor; Vetoquinol B.V., Breda, the Netherlands; 0.25 mg/kg) and ketamine (Narketan; Vetoquinol B.V.; 40 mg/kg); 0.02 mg/kg glycopyrronium (Robinul; Chiesi Pharmaceuticals GmbH, Vienna, Austria) was injected subcutaneously to reduce bronchial secretion. First, click-evoked ABRs were recorded to confirm normal hearing (for details, see Ramekers et al. 2014). Thresholds around 40 dB peak equivalent SPL (peSPL) were considered to indicate normal hearing. An incision was made starting approximately 20–25 mm rostral to bregma, proceeding caudally, and to the right behind the right pinna, ending approximately 5 mm ventrocaudal to the pinna. Six transcranial head screws were positioned for head connector anchoring and for active or reference electrodes for ABR or eCAP recordings. The head connector of the electrode array was positioned approximately on bregma and fixed with cold-curing dental cement (ProBase Cold; Ivoclar Vivadent AG, Schaan, Liechtenstein), after insertion of the electrode array into the cochlea. In order to reach the cochlea, the right pinna was folded rostrally and the bulla was exposed. A hole was hand-drilled in the bulla just large enough to visualize the cochlear round window niche and part of the basal turn. In the basal turn of the cochlea, within 1 mm from the round window, a 0.4-mm cochleostomy was hand-drilled into the scala tympani. A custom-made three-contact electrode array (MED-EL GmbH, Innsbruck, Austria) was inserted approximately 4 mm into the cochleostomy, after which brief electrode impedance measurements and eCAP recordings were performed to verify proper functioning and positioning of the array. The electrode lead was fixed to the bulla with dental cement (GC Fuji PLUS; GC Corporation, Tokyo, Japan), and finally, the head connector was fixed to the skull and the wound was closed with two layers of absorbable sutures. Before antagonizing, the dexmedetomidine-induced anesthesia with atipamezole (Atipam; Dechra Pharmaceuticals, Northwich, UK; 1 mg/kg), carprofen (Carporal; AST Farma, Oudewater, the Netherlands; 4 mg/kg) and the non-ototoxic antibiotic enrofloxacine (Baytril; Bayer AG, Leverkusen, Germany; 5 mg/kg) were injected subcutaneously.

### Deafening

The animals were systemically deafened 4 weeks after cochlear implantation, as previously described (Ramekers et al. 2014, 2015a). After induction of anesthesia (0.25 mg/kg dexmedetomidine and 40 mg/kg ketamine i.m.), click-evoked ABRs were recorded in order to assess changes in hearing thresholds as a result of the implantation surgery and presence of the intracochlear electrode array. Deafening was done by subcutaneous injection of kanamycin (Sigma-Aldrich, St. Louis, MO, USA; 400 mg/kg) and subsequent infusion of furosemide (Centrafarm, Etten-Leur, the Netherlands; 100 mg/kg) into the external jugular vein within 45–60 min after kanamycin injection.

### eCAP Recordings

Except for the first recording session immediately after the implantation surgery, when the animals were still anesthetized (day -28), all eCAPs were recorded while the animals were awake. On day 0, this meant that eCAPs were recorded immediately prior to induction of anesthesia for the deafening surgery. Apart from during these eCAP recording sessions, the animals did not receive any additional electrical stimulation via their CI electrodes.

A recording session typically took 10–15 min, during which the animal was fixed into a comfortable cloth restraining sock, which still allowed continuous visual inspection of the animal’s wellbeing. A 50-cm custom-made shielded cable connected the head connector of the electrode array with a MED-EL PULSARci^100^ cochlear implant, which was controlled using custom MATLAB scripts (version 7.11.0; MathWorks, Natick, MA, USA) via a Research Interface Box 2 (Department of Ion Physics and Applied Physics, University of Innsbruck, Innsbruck, Austria) and a National Instruments data acquisition card (PCI-6533; National Instruments, Austin, TX, USA).

Both stimulation and recording were done with monopolar configuration; with few exceptions, the most apical of the three electrode contacts was used for stimulation, and the most basal one for recording. Two head bolts positioned approximately 8 mm to either side of bregma were used as stimulation reference (ipsilateral) and recording reference (contralateral to the implanted ear). Biphasic current pulses were presented with alternating polarity to reduce stimulation artifact, whereas recordings to a subthreshold stimulus were subtracted to eliminate measurement onset artifacts.

**Fig. 1.**
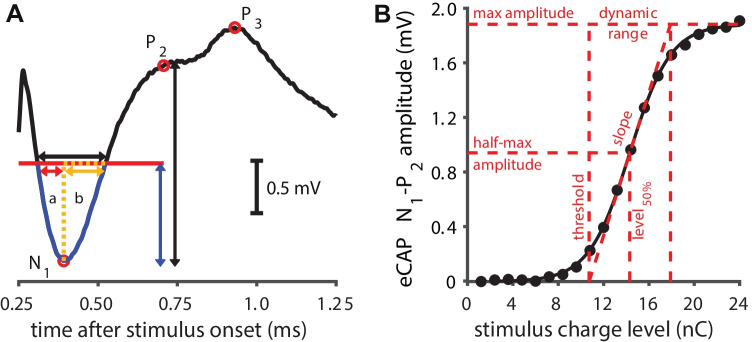
Analysis of the eCAP. **A** For the main analysis in this study the N_1_-P_2_ amplitude (vertical black double arrow) and the N_1_ latency were used. In addition, the eCAP peak width and peak area were determined at half of the N_1_-P_2_ amplitude (blue double arrow/red horizontal line). The full peak width was defined as the length of the red line between the two crossings with the eCAP waveform (black horizontal double arrow), and the full peak area as the area enclosed between the red line and the blue portion of the eCAP waveform. Both the peak width and the peak area were divided into two (asymmetrical) halves by the vertical dashed orange line drawn upward from the N_1_ peak. Note that only the first 1 ms of the eCAP recording is shown. **B** The eCAP N_1_-P_2_ amplitude was used to construct amplitude growth functions (AGFs) which we subsequently fitted with a Boltzmann sigmoid function. The resulting eCAP characteristics (shown in red) were compared between and across animals over time

**Fig. 2:**
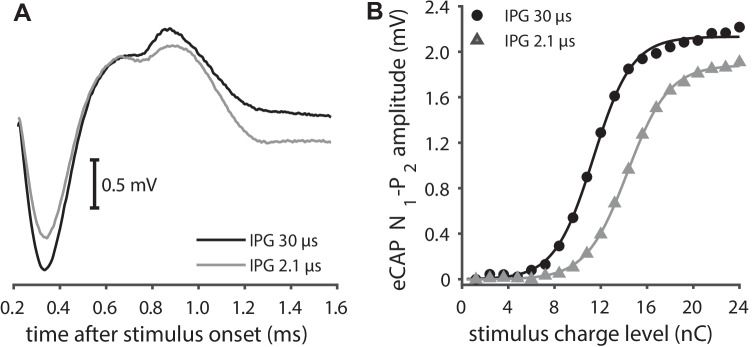
Illustration of IPG effects. **A** Two eCAP examples from a single normal-hearing animal. The stimulus IPG was 2.1 µs for the grey eCAP trace, and 30 µs for the black eCAP trace. All other stimulation parameters (including phase duration of 50 µs and the maximum charge level of 24 nC) and recording parameters were identical for these two examples. Increasing the IPG clearly leads to an increase in eCAP amplitude. **B** Two AGFs corresponding to the eCAPs in panel **A**. These AGFs resulted from N_1_-P_2_ amplitudes from eCAPs evoked using charge levels increasing from 1.2 to 24 nC in 20 linear (equidistant) steps. The sigmoidal curves represent the Boltzmann fit through all 20 data points (Eq. 1). From these sigmoidal curves, the decrease in threshold and level_50 %_, as well as the increase in maximum amplitude and AGF slope with increasing IPG, can be clearly observed (see also Fig. 1B)

In order to verify that intended current levels were within compliance limits, electrode impedances were measured and evaluated at the beginning and end of every session. The eCAP amplitude was defined as the voltage difference between the eCAP N_1_ and P_2_ peaks (Fig. 1A). AGFs were subsequently constructed using 20 linearly spaced current steps (Fig. 1B), typically from 24 to 480 µA (or 1.2 to 24 nC, since the pulse phase duration was 50 µs). The AGFs were recorded once with an IPG of 2.1 µs (the shortest IPG possible with the MED-EL PULSAR implant), and once with an IPG of 30 µs (the longest IPG possible), examples of which are shown in Fig. 2A. Current levels were randomly permuted to prevent adaptation of the neural response, and to optimize the animal’s comfort. Within this permutation order, each current level was presented 25 times for both stimulus polarities at a stimulation rate of 50 Hz. The recording sample frequency was 1.2 MHz, so that the ~1.7-ms eCAP recording, starting after 250–300-µs measurement delay (blanking time) relative to stimulus onset, consisted of 2048 samples.

### Termination and Histological Analysis

After the final awake recording session on day 49, ABRs were recorded for the third time as described above, and the animals were anesthetized (0.25 mg/kg dexmedetomidine and 40 mg/kg ketamine i.m.) before termination with an intracardial overdose of pentobarbital (Euthanimal; Alfasan B.V., Woerden, the Netherlands). Both the right implanted and the left untreated cochleas were harvested and processed for light microscopical analysis as described previously (de Groot et al. 1987; van Loon et al. 2013; Ramekers et al. 2020). In brief, intra-labyrinthine cochlear fixation was achieved with a fixative of 3 % glutaraldehyde, 2 % formaldehyde, 1 % acrolein, and 2.5 % DMSO in a 0.08 M sodium cacodylate buffer. The cochleas were then decalcified in 10 % EDTA, secondarily fixed in 1 % osmium tetroxide and 1 % potassium ruthenium cyanide, and embedded in Spurr’s low-viscosity resin. Semi-thin (1-µm) mid-modiolar sections were stained with 1 % methylene blue, 1 % azur B, and 1 % borax.

**Fig. 3:**
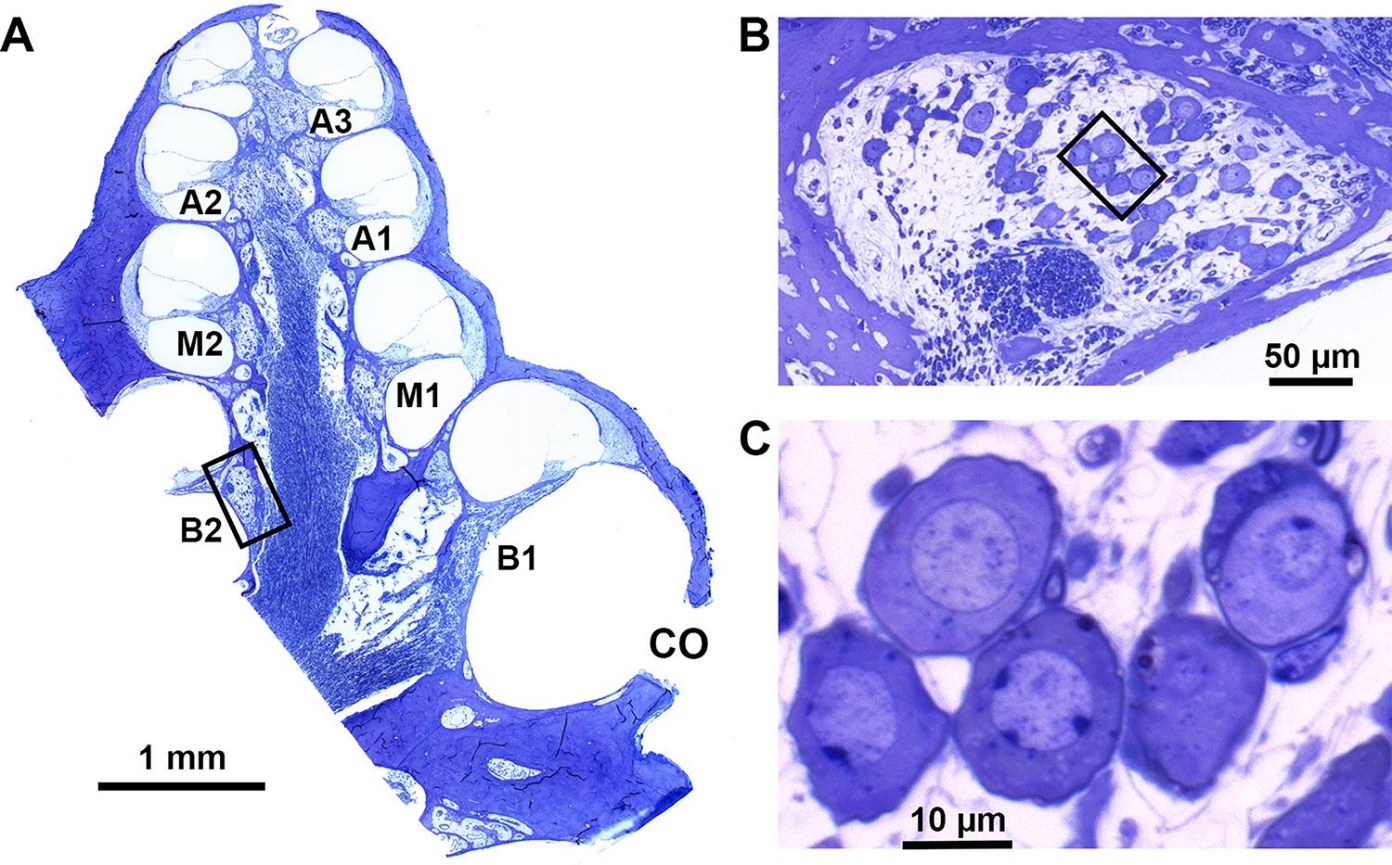
**A** Spiral ganglion cell packing densities were determined in seven transections of Rosenthal’s canal (B1-A3) in a standardized midmodiolar plane. Black rectangle indicates location of panel B. **B** Rosenthal’s canal containing the spiral ganglion cells in the second basal turn (B2). Black rectangle indicates location of panel C. **C** Close-up of several type I spiral ganglion cells

With a Leica DFC450 C digital camera mounted on a Leica DMRA light microscope and a 40× oil immersion lens (Leica Microsystems GmbH, Wetzlar, Germany), micrographs of each transection of Rosenthal’s canal were obtained (2 basal, 2 middle, and 3 apical transections; see Fig. 3A). The number of type I and type II SGCs and the size (perikaryal area) of the type I SGCs were determined using ImageJ (version 1.52a; National Institutes of Health, Bethesda, MA, USA), and the SGC packing density in Rosenthal’s canal was calculated for each of the seven transections separately by dividing the SGC count by the cross-sectional area of the respective transection (e.g., Fig. 3B, C). In these mid-modiolar sections, we additionally determined survival of both inner (IHC) and outer hair cells (OHC).

### eCAP Analysis

Primary eCAP analysis was done in accordance with our previous studies (Ramekers et al. 2014, 2015a; Vink et al. 2020). For the main results (absolute eCAP characteristics and accompanying IPG effects), the eCAP N_1_-P_2_ amplitude was determined for each current level (Fig. 1A), so that an input-output curve or AGF could be constructed (Fig. 1B). A Boltzmann sigmoid function was fitted through the 20 resulting data points:

1 $${V}_{eCAP}=A+\frac{B}{1+{e}^{-\frac{I-C}{D}}} ,$$

where *I* is the input current level, *V*_*eCAP*_ is the recorded eCAP N_1_-P_2_ output voltage, and *A*–*D* are fitting parameters. *A* represents the noise level, *B* is the maximum eCAP N_1_-P_2_ amplitude, *C* is the current level at the inflection point of the sigmoid (at the half-maximum eCAP amplitude), and *D* relates to the dynamic range. Five eCAP characteristics were derived from these four fitting parameters: maximum eCAP amplitude in mV (*B*), level_50%_ in dB re 1 nC (*C*), dynamic range in dB (4 × *D*), slope in mV/nC (*B* / [4 × *D*]), and threshold in dB re 1 nC (*C* − 2 × *D*). Note that level_50%_, slope, and threshold are evaluated in terms of charge rather than current in order to increase comparability with our previous studies in which multiple phase durations were used. A sixth eCAP characteristic we evaluated was the eCAP N_1_ latency, defined as the mean time difference between the stimulus onset and the N_1_ peak for the eCAPs evoked by the three highest current levels used, and expressed in milliseconds.

IPG effects were calculated from the difference in fitting parameters *A*–*D* between AGFs constructed with 30-µs IPG eCAPs and 2.1-µs IPG eCAPs (Fig. 2B). The IPG effect for maximum eCAP amplitude (Δamplitude) was calculated as the difference between the two IPG conditions (IPG 30 µs minus IPG 2.1 µs) divided by the maximum eCAP amplitude for the 30-µs IPG condition; Δslope as the absolute difference between the two IPG conditions; and Δthreshold, Δdynamic range, and Δlevel_50%_ as the difference between the two conditions in dB. The IPG effect for latency (Δlatency) was calculated as the latency difference between the two conditions (IPG 30 µs minus IPG 2.1 µs).

**Fig. 4:**
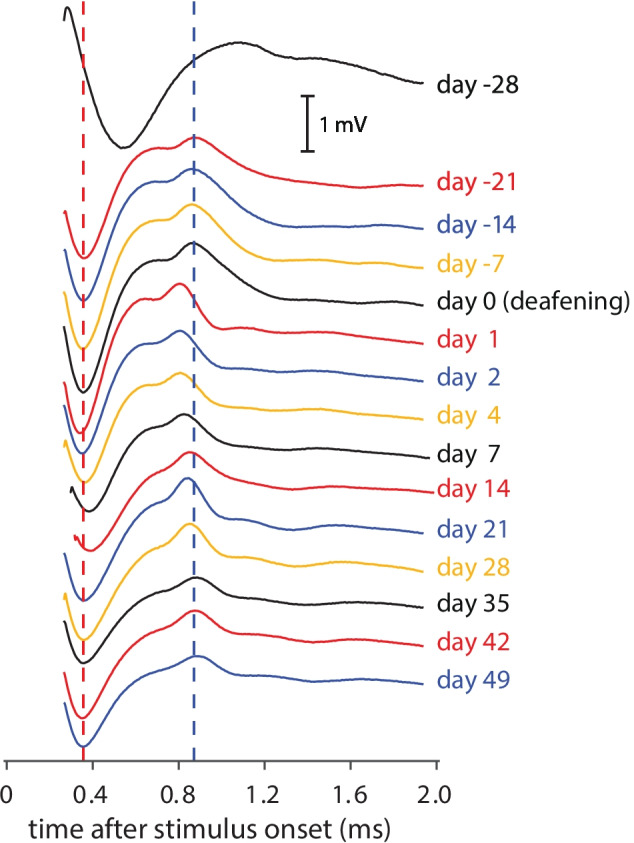
eCAPs over time after implantation in a single animal. Stimulation conditions were similar for all 15 eCAPs (30 µs IPG; highest current level); only the measurement delay varied (250–300 µs) in order to overcome clipping artifacts due to amplifier saturation. The vertical dashed red line indicates the mean N_1_ latency pre-deafening (days -21 to day 0); the dashed blue line indicates the mean P_2_ latency pre-deafening (days -21 to day 0). Note that the first recording (day -28) was performed immediately upon implantation while the animal was still anesthetized, whereas all other recording were performed in the awake condition

Secondarily, the eCAP N_1_ peak width and peak area were evaluated for pulses with 50-µs phase duration and 30-µs IPG at the highest current level applied, at half the N_1_-P_2_ amplitude (red horizontal line in Fig. 1A). This analysis was done since the morphology of the eCAP waveform is known to change after deafening (e.g., Ramekers et al. 2015a; Strahl et al. 2016), and was performed in two different ways. In case the start of the recording was before the first crossing of the red line with the eCAP, both peak widths (red double arrow for first half; orange double arrow for second half) and both peak areas *a* and *b* were calculated, as well as the total peak width (black double arrow) and total peak area (*a* + *b*). In case the recording started after this first crossing (because of relatively short N_1_ latency, or relatively long measurement delay), only the second half of the N_1_ peak (i.e., orange double arrow and peak area *b*) could be determined. In order to be able to directly compare these various measures, both within and across animals, they were normalized by dividing by the mean normal-hearing values from days -21, -14, -7, and 0. The data from day -28 were excluded from these reference means, because the eCAPs recorded directly after implantation deviated in several aspects from all those subsequently recorded (see examples in Fig. 4).

### Correlations Between IPG Effects and SGC Survival

Correlations between eCAP IPG effects and SGC survival were assessed using the mean SGC packing density across all seven cochlear turns. For three reasons, the cochlear mean was chosen rather than only the basal turn where the electrode array was located: (1) since the eCAP AGFs reached an upper asymptote, we assumed virtually that all SGCs contribute to the eCAP; (2) we have previously reported that the correlation between these IPG effects and SGC packing density for basal, middle, and apical locations separately is roughly similar (Fig. 8 in Ramekers et al. 2014); and (3) using only the basal SGC packing density in the present data set yielded lower correlation coefficients, simply because of the larger variability that is inherent to taking a smaller sample.

### Statistical Analysis

Changes in ABR threshold after implantation (day − 28 versus day 0) as well as differences in hair cell presence between ears after deafening were assessed with non-parametric Wilcoxon signed-rank tests. Differences in SGC survival and mean perikaryal area across the seven cochlear locations and between implanted right ears and non-implanted left ears were tested for statistical significance using linear mixed model (LMM) analysis. Cochlear location (in relative distance from the round window as described by van Loon et al. 2013) was treated as a covariate and ear (right implanted versus left) as a factor. Changes in eCAP measures over time after implantation (day -21 to day 0; four time points) and over time after deafening (day 1 to day 49; 10 time points) were analyzed with rmANOVA. Simple contrasts were used to assess from which time point onward eCAP measures did not significantly differ from the final data point at day 49.

## Results

### Effects of Implantation and Deafening on Acoustic Hearing

**Fig. 5:**
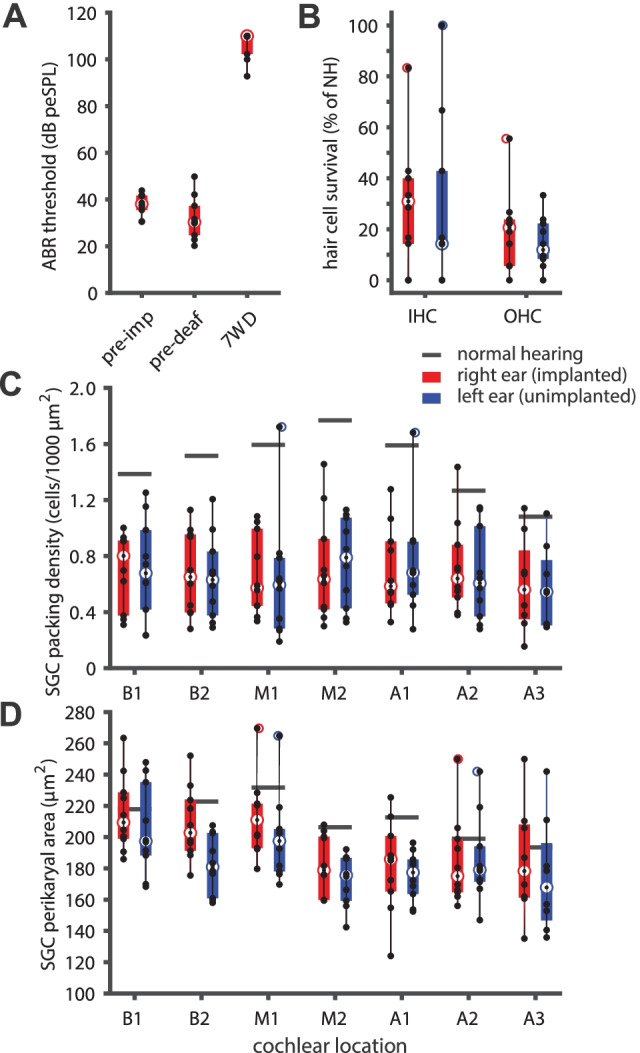
**A** Individual click-evoked ABR thresholds before implantation (“pre-imp”), 4 weeks after implantation (immediately prior to deafening; “pre-deaf”), and 7 weeks after deafening (“7WD”). Box plots indicate the median thresholds and the 25th and 75th percentiles; whiskers indicate the extreme values that are not considered to be outliers. *N* = 10 animals. **B** Hair cell survival sampled in the same midmodiolar sections as were used for SGC quantification. Box plots indicate median hair cell survival and the 25th and 75th percentiles; whiskers indicate the extreme values that are not considered to be outliers. There were no statistically significant differences between the right implanted (red) and left non-implanted ears (blue). *N* = 10 animals. IHC, inner hair cell; OHC, outer hair cell; NH, normal-hearing. **C** Spiral ganglion cell packing density across all seven cochlear locations for the implanted right (red) and the non-implanted left ears (blue) in individual animals. Box plots indicate median SGC survival and the 25th and 75th percentiles; whiskers indicate the extreme values that are not considered to be outliers. Mean normal-hearing values are indicated by the grey horizontal lines. There were no statistically significant differences in SGC survival between ears or across locations. *N* = 10 animals. **D** Spiral ganglion cell size across all seven cochlear locations for the implanted right (red) and the non-implanted left ears (blue) in individual animals. Box plots indicate median SGC perikaryal area and the 25th and 75th percentiles; whiskers indicate the extreme values that are not considered to be outliers. Normal-hearing values are indicated by the grey horizontal lines. SGCs were significantly larger in the base than in the apex, and larger in the right implanted ears than in the left non-implanted ears. *N* = 10 animals

ABR thresholds were assessed prior to implantation, 4 weeks later before deafening, and again 7 weeks later before euthanasia. Before implantation, all animals had normal ABR thresholds (median 38 dB peSPL). Cochlear implantation did not lead to threshold elevation (median 30 dB peSPL), which was confirmed with a non-parametric Wilcoxon signed-rank test (*Z* = -1.6, *P* = 0.11). After deafening, all animals had a substantial threshold shift with a median of 75 dB (range 57.9–87.2 dB) (Fig. 5A).

Hair cell survival 7 weeks after deafening was reduced to 17 % of that in normal-hearing animals, with a moderate discrepancy between inner hair cell (median of right implanted ears 31 %; median of left non-implanted ears 14 %) and outer hair cell survival (median of right implanted ears 21 %; median of left non-implanted ears 12 %) (Fig. 5B). Non-parametric Wilcoxon signed-rank tests revealed that there were no significant differences in survival within-subject between the implanted right and the non-implanted left ear (inner hair cells *Z* = -0.42, *P* = 0.68; outer hair cells *Z* = -1.25, *P* = 0.21).

### Spiral Ganglion Cell Histology

SGC survival at the end of the experimental period (day 49) was assessed by evaluating packing density and size (perikaryal area) across all seven transections of Rosenthal’s canal in both cochleas (see Fig. 3). The SGC packing densities shown in Fig. 5C demonstrate the substantial degeneration that had occurred during the 7 weeks following systemic deafening (relative to NH controls from previous studies [Ramekers et al. 2014, 2015a] shown in horizontal black lines per cochlear location). Across the entire cochlea, mean SGC survival was highly similar between the right implanted (46.7 % of NH controls) and the left non-implanted ears (46.5 % of NH controls). Linear mixed model analysis confirmed that there was no difference in packing density between ears [*F*_(1,46)_ = 0.001; *P* = 0.98], no differences across cochlear locations [*F*_(1,64)_ = 0.20; *P* = 0.66], and no interaction between the two [*F*_(1,64)_ = 0.02; *P* = 0.90].

Mean perikaryal area was moderately reduced by deafening (Fig. 5D), and was slightly smaller for the left non-implanted ears (87.3 % of NH controls) than for right implanted ears (92.4 % of NH controls). Linear mixed model analysis revealed that the difference in SGC size between ears was statistically significant [*F*_(1,50)_ = 5.9; *P* = 0.019]. In addition, SGC perikaryal area significantly varied with cochlear location [*F*_(1,73)_ = 20.3; *P* < 0.001], confirming that basal SGCs are larger than apical SGCs; no significant interaction between ear and location was observed [*F*_(1,73)_ = 1.3; *P* = 0.25].

Taken together, these results suggest that neither the insertion/presence of the intracochlear electrode array nor the brief exposure to electrical current during weekly eCAP recording sessions caused any SGC loss in addition to the bilateral deafness-induced degeneration. However, slightly larger cells in the implanted ears may indicate preservation of cell size as a result of electrical activation of otherwise largely quiescent auditory nerve.

### Absolute eCAP Measures

eCAP waveforms from a single animal and for all 15 recording sessions are shown in Fig. 4. For all animals, the eCAPs at day -28 had longer latencies and higher thresholds, which may have been a result of anesthesia and/or of acute (transient) effects of electrode insertion. For all further (statistical) analyses, the data collected at day -28 were therefore excluded. From day -21 to day 0, the eCAP morphology was typically highly similar. Instant changes were observed after deafening (from day 1 onward), which were most clearly manifest in terms of shorter latencies. Whereas eCAP latencies returned to normal-hearing values typically within 2–3 weeks, a more gradual but permanent change in eCAP morphology was that the amplitude became smaller – an observation that coincided with, and was likely a result of, the loss of SGCs. Note that the measurement delay of 250 µs was occasionally increased up to 300 µs, in order to overcome clipping artifacts due to amplifier saturation (e.g., day 7 and day 14 in Fig. 4). This increase was often necessary shortly after deafening, and it regularly (but not consistently) coincided with longer eCAP latencies.

**Fig. 6:**
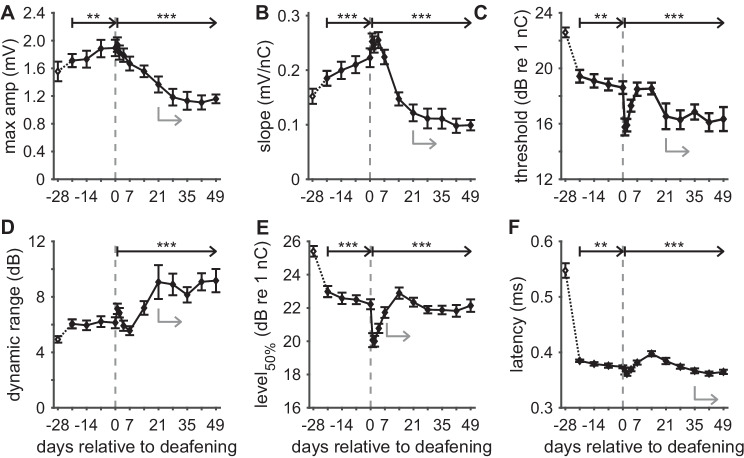
eCAP characteristics over time after implantation and subsequent deafening for 30-µs IPG pulses. **A** Maximum eCAP amplitude; **B** steepest slope of the amplitude growth function; **C** eCAP threshold; **D** dynamic range of the amplitude growth function; **E** level_50%_ (the charge level needed to attain the half-maximum eCAP amplitude); **F** eCAP N_1_ latency. Open symbols at day -28 indicate that these data may deviate from subsequent data because they were recorded directly upon cochlear implantation, while the animals were still under anesthesia. Dashed vertical lines indicate time of deafening. Asterisks indicate statistically significant main effect of rmANOVA (details in Table 1): *P < 0.05; **P < 0.01; ***P < 0.001. Grey arrows indicate the time points beyond which the data did not significantly deviate from the final data point at day 49, as assessed with simple contrasts (*P* > 0.05). *N* = 9 for days 2 and 35; *N* = 8 for days 42 and 49; *N* = 10 animals for the remaining 11 time points; error bars represent SEM

For all 15 time points, the eCAP was analyzed as illustrated in Fig. 1, and parameterized by six primary characteristics of which mean values across animals are shown in Fig. 6 (A: maximum amplitude; B: AGF slope; C: threshold; D: dynamic range; E: level_50%_; and F: latency). As for the example in Fig. 4, the eCAP N_1_ latency for the first (anesthetized) recording at day -28 was a clear outlier for all 10 animals (Fig. 6F). Similarly, the threshold (Fig. 6C) and level_50%_ (Fig. 6E), which can be interpreted as the median excitation threshold for individual SGCs, were substantially higher at day -28 than at any later time point. Statistical analyses were therefore performed for data from day -21 onward: for the assessment of effects of implantation over time in the normal-hearing animal, days -21 to 0 were compared; and for the assessment of subsequent deafness-induced changes, days 1 to 49 were compared.

In the normal-hearing animals, all eCAP characteristics varied slightly but statistically significantly with time after implantation from day -21 onward, except for the dynamic range (Fig. 6D) (see Table 1; “pre-deafening”). A gradual increase in maximum eCAP amplitude and AGF slope and a subtle decrease in threshold, level_50 %_ and latency together suggest that SGC excitability increased during the first 4 weeks after electrode insertion.

**Table 1:** rmANOVA results for absolute eCAP characteristics

	**Pre-deafening (*N* = 10)**			**Post-deafening (*N* = 8)**		
	*F*	*df*	*P*	*F*	*df*	*P*
**Amplitude**	6.7	3, 27	0.0016	34.9	9, 63	< 0.001
**Slope**	11.9	3, 27	< 0.001	46.7	9, 63	< 0.001
**Threshold**	5.5	3, 27	0.0044	5.4	9, 63	< 0.001
**Dynamic range**	0.87	3, 27	0.47	8.1	9, 63	< 0.001
**Level**_**50%**_	8.5	3, 27	< 0.001	12.9	9, 63	< 0.001
**Latency**	6.4	3, 27	0.0021	12.3	9, 63	< 0.001

After deafening, all six eCAP characteristics highly significantly changed with time (see Table 1; “post-deafening”). Two distinct patterns of these changes over time were observed: (1) the maximum amplitude and AGF slope decreased monotonically, while (2) the other four characteristics showed an initial peak around days 1–2 (negative for threshold, level_50%_, and latency; positive for the dynamic range), followed by a peak in the opposite direction around days 7–14, and finally a second reversal, decaying into a relatively stable state. All six eCAP characteristics had stabilized before day 49, as assessed with simple contrast analysis, and illustrated with grey arrows in Fig. 6.

### IPG Effects

**Fig. 7:**
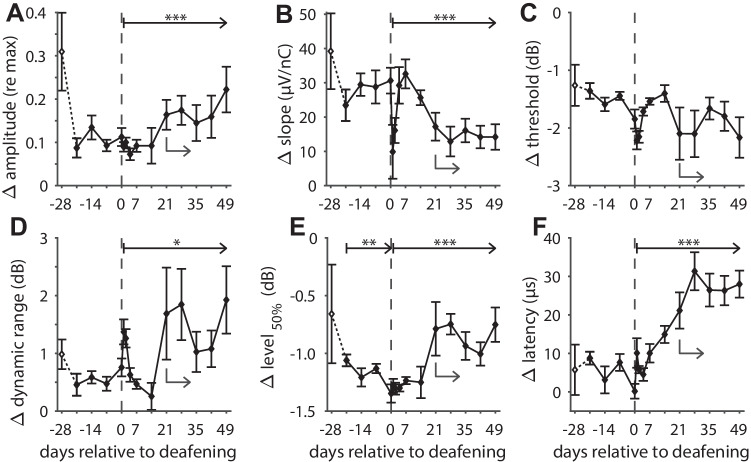
As Fig. 6, but with IPG effects instead of absolute eCAP characteristics over time after implantation and subsequent deafening. **A** Difference in maximum eCAP amplitude when the IPG is increased from 2.1 to 30 µs, relative to the largest of the two (i.e., 30-µs IPG); **B** difference in steepest slope of the amplitude growth function; **C** difference in eCAP threshold; **D** difference in dynamic range of the amplitude growth function; **E** difference in level_50%_ (the charge level needed to attain the half-maximum eCAP amplitude), occasionally also referred to as the IPG offset; **F** difference in eCAP N_1_ latency. Open symbols at day -28 indicate that these data may deviate from subsequent data because they were recorded directly upon cochlear implantation, while the animals were still under anesthesia. Dashed vertical lines indicate time of deafening. Asterisks indicate statistically significant main effect of rmANOVA (details in Table 2): *P < 0.05; **P < 0.01; ***P < 0.001. Grey arrows indicate the time points beyond which the data did not significantly deviate from the final data point at day 49, as assessed with simple contrasts (*P* > 0.05). *N* = 9 for days 0, 2, and 35; *N* = 8 for days 42 and 49; *N* = 10 animals for the remaining ten time points; error bars represent SEM

eCAPs were recorded in response to biphasic current pulses with either a 2.1-µs or a 30-µs IPG. The difference in eCAP characteristics between the two conditions (referred to as “IPG effects”; see Fig. 2) over time after implantation is shown in Fig. 7, analogous to the absolute eCAP measures in Fig. 6.

In contrast to the absolute eCAP measures, the IPG effects did not significantly vary for five of the six eCAP characteristics before deafening (see Fig. 7A–D, F and Table 2; pre-deafening). The only statistically significant change before deafening was a slight increase from 1.1 to 1.4 dB for Δlevel_50%_ (Fig. 7E). Hence, IPG effects may be considered more robust over time than absolute eCAP measures, although the IPG effects for especially the maximum amplitude, AGF slope, and level_50%_ for the first recordings at day -28 were equally affected by anesthesia or by acute effects of electrode insertion. After deafening, all IPG effects except for that of threshold (Fig. 7C) significantly changed over time (see Table 2; post-deafening). For maximum amplitude (Fig. 7A), dynamic range (Fig. 7D) and latency (Fig. 7F) the IPG effects increased with duration of deafness, while for the AGF slope (Fig. 7B) and level_50%_ (in absolute terms) (Fig. 7E), the IPG effects became smaller after deafening. All six eCAP IPG effects had in common that they had stabilized before the end of the experimental period at day 49, evidenced by the absence of significant differences from the final time point for those from day -21 onward (simple contrasts; grey arrows in Fig. 7).

**Table 2:** rmANOVA results for eCAP IPG effects

	**Pre-deafening (*N* = 9)**			**Post-deafening (*N* = 8)**		
	*F*	*df*^*a*^	*P*	*F*	*df*	*P*
**Amplitude**	2.8	3, 24	0.064	2.6	9, 63	0.014
**Slope**	1.5	3, 24	0.25	2.7	9, 63	0.010
**Threshold**	2.6	3, 24	0.072	1.1	9, 63	0.40
**Dynamic range**	0.97	1.8, 14	0.42	1.6	9, 63	0.15
**Level**_**50%**_	5.2	3, 24	0.0063	3.5	9, 63	0.0015
**Latency**	2.2	3, 24	0.12	12.4	9, 63	< 0.001

^a^Greenhouse-Geisser correction applied in case assumption of sphericity was violated

### Correlation Between eCAPs and Histology

**Fig. 8:**
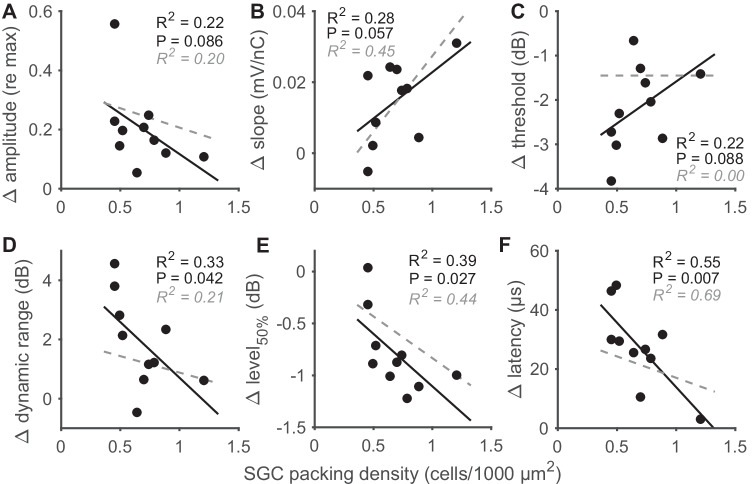
Correlations between IPG effects on various eCAP measures (see Fig. 7) and spiral ganglion cell survival for all ten animals. eCAPs were recorded on experimental day 49, several hours before euthanasia and fixation of cochlear tissue for histological processing. **A** Difference in maximum eCAP amplitude when the IPG is increased from 2.1 to 30 µs, relative to the largest of the two (i.e., 30-µs IPG); **B** difference in steepest slope of the amplitude growth function; **C** difference in eCAP threshold; **D** difference in dynamic range of the amplitude growth function; **E** difference in level_50%_ (the charge level needed to attain the half-maximum eCAP amplitude); **F** difference in eCAP N_1_ latency. The black trend lines indicate the relation between IPG effects and SGC packing density from the animals in the present study. For comparison, the grey dashed lines indicate the relation between the same measures from one of our previous studies (Ramekers et al. 2014); the corresponding *R*^2^ values are given in each panel in grey italic font

IPG effects have been shown to correlate with SGC survival across animals (Prado-Guitierrez et al. 2006; Ramekers et al. 2014, 2015a; Schvartz-Leyzac et al. 2020a, b; Vink et al. 2020). Since SGC quantification is not possible within-subject over time, only the IPG effects at the final day 49 could be rationally compared to the SGC histology. In Fig. 8, IPG effects for all 10 animals are plotted as function of the mean SGC packing density across the implanted right cochlea (A: Δmaximum amplitude; B: ΔAGF slope; C: Δthreshold; D: Δdynamic range; E: Δlevel_50%_; F: Δlatency). Even though all animals were treated identically, there was a notable spread in SGC survival across animals, which correlated well with the six IPG effects (*R*^2^ between 0.22 and 0.55). Furthermore, with the exception of threshold, all IPG effects exhibited a similar relation with SGC packing density (black solid lines) as for a group of 18 acutely implanted animals in a previous study (grey dashed lines; Ramekers et al. 2014).

### Electrode Impedances and eCAP Measures

**Fig. 9:**
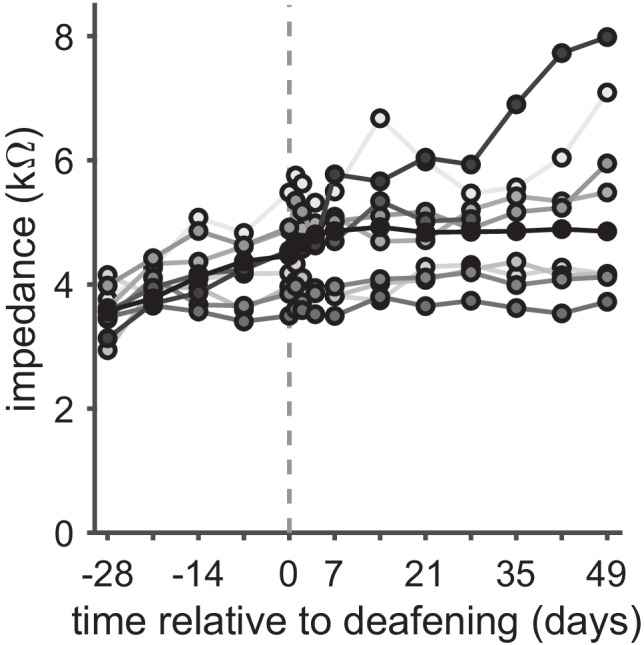
Stimulation electrode impedances over time after cochlear implantation. Lines represent individual animals. Grey scale of lines and symbols indicates order of mean eCAP thresholds over time: lightest grey indicates lowest mean threshold; black indicates highest mean threshold

Stimulation electrode impedances were measured before and after each eCAP recording session, the latter of which was used for subsequent analysis. In Fig. 9, these impedances are shown over time after implantation for each animal individually. Grey scales for lines and symbols indicate the order of mean eCAP thresholds across all time points: lightest grey represents the animal with lowest mean threshold; black indicates the animal with the highest mean threshold. Initial impedances upon implantation were similar across animals (approximately 3–4 kΩ), and in general, there was a gradual increase over the course of the following 11 weeks. In four animals, however, there hardly seemed to be any change in impedance over time. Seemingly randomly ordered grey-scaled lines (light to dark reflecting low to high eCAP thresholds) suggest that neither absolute impedances nor the extent of impedance increase appeared to be indicative of the eCAP threshold.

In order to rule out any confounding effects of these diverging impedances over time, we assessed whether any of the previously presented eCAP measures were correlated with the impedances at days 0 (normal-hearing) and 49 (7 weeks deaf). The *R*^*2*^ and *P* values for the absolute eCAP characteristics are presented in Table 3; those for the eCAP IPG effects are shown in Table 4. None of the 12 eCAP measures were statistically significantly dependent on electrode impedance at either of the two tested time points.

**Table 3:** Correlations between absolute eCAP characteristics and electrode impedances

	**Day 0 (NH)**		**Day 49 (deaf)**	
	*R*^*2*^	*P*	*R*^*2*^	*P*
**Amplitude**	0.18	0.23	0.03	0.66
**Slope**	0.01	0.77	0.03	0.62
**Threshold**	0.24	0.15	0.19	0.21
**Dynamic range**	0.17	0.23	0.35	0.069
**Level**_**50%**_	0.15	0.28	0.00	0.98
**Latency**	0.003	0.89	0.01	0.74

**Table 4:** Correlations between eCAP IPG effects and electrode impedances

	**Day 0 (NH)**		**Day 49 (deaf)**	
	*R*^*2*^	*P*	*R*^*2*^	*P*
**Amplitude**	0.03	0.66	0.06	0.52
**Slope**	0.04	0.61	0.14	0.29
**Threshold**	0.00	0.99	0.20	0.20
**Dynamic range**	0.00	0.92	0.28	0.12
**Level**_**50%**_	0.03	0.65	0.32	0.092
**Latency**	0.06	0.52	0.12	0.34

### eCAP Peak Width and Area

**Fig. 10:**
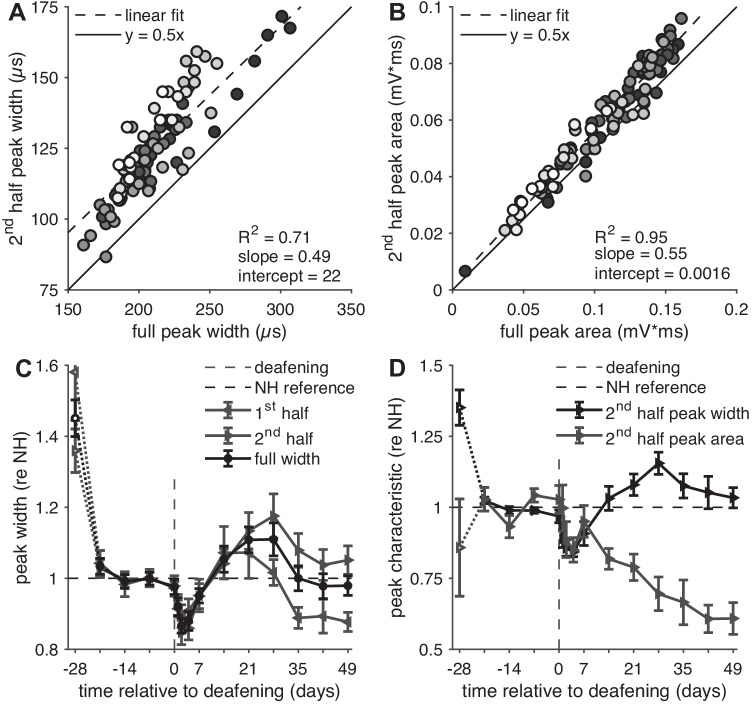
N_1_ peak width and peak area. **A** Second half of the peak width (see Fig. 1A) as function of the full peak width for all available animals/recording sessions. Black circles indicate first recording session at day -28, white circles indicate the last session at day 49, and grey circles are intermediate sessions. From a maximum of 150 symbols (10 animals times 15 recording sessions), 107 pairs are shown since the first half of the N_1_ peak (and thus the full peak as well) could not be measured in the remaining 43 sessions. The diagonal (*y* = 0.5*x*) signifies a theoretical symmetrical peak, in which the full peak width is twice that of the second half. With practically all data above this diagonal, the eCAP N_1_ peak appears to be asymmetrical, with a steeper falling edge than the subsequent rising edge. Lighter shades appear to be positioned farther from the diagonal, suggesting more pronounced asymmetry after deafening. **B** As **A**, but with peak area instead of peak width. The two halves of the N_1_ peak area are more balanced than their widths, as evidenced by the closer location of the data to the diagonal. **C** The mean values of the first half, second half, and full peak width across animals, plotted over time after implantation. All data are normalized to the normal-hearing values (mean from days -21 to 0) for each animal individually; only data are shown when all three measures were available for individual animals and time points (*N* ranges from 5 to 10 animals across time points). In the first week after deafening, all three measures show that the N_1_ peak becomes rapidly but transiently narrower. Subsequently, all three measures are gradually widening, with a peak around 2–4 weeks after deafening, after which the first and second half begin to diverge. In the last two weeks, the first half of the N_1_ peak is clearly shorter than normal, whereas the second half is slightly longer. Open symbols at day -28 indicate that these data may deviate from subsequent data because they were recorded directly upon cochlear implantation, while the animals were still under anesthesia. Dashed vertical lines indicate time of deafening; error bars represent SEM. **D** As **C**, but peak width and peak area both plotted for only the second half of the N_1_ peak. All available data are shown (*N* = 10 animals)

eCAP peak width and area were evaluated as secondary outcome measures to examine whether eCAP morphology has added value to the eCAP amplitude and latency in explaining the SGC degeneration process over time. The eCAP examples in Fig. 4 illustrate that the full peak width and full peak area (see Fig. 1A) cannot always be determined; we therefore additionally included the width and area of the second half (i.e., from the minimum to the point halfway in between the N_1_-P_2_ amplitude) for subsequent analysis (Fig. 10). In roughly two-thirds of the available eCAPs, the full peak width and area could be determined; in all 150 instances could the second half be reliably determined.

Figure 10A, B indicates that since the first half of the eCAP N_1_ peak cannot always be determined, using only the second half is a valid and relatively accurate alternative. In case of peak width, there is a systematic overestimation of the full width when only the second half is available (indicated by an intercept of 22 µs in Fig. 10A), which shows that the eCAP N_1_ peak is not symmetrical but rather has a steeper initial falling edge than the subsequent rising edge. In case of peak area, however, the second half more accurately predicts the full peak (Fig. 10B) than for peak width.

All three measures of peak width (i.e., full width and both halves) followed the same pattern over time as previously shown for the eCAP threshold, level_50%_, and – most notably – N_1_ latency (Fig. 6): a sharp decrease directly after deafening, followed by two reversals within several weeks (Fig. 10C). Interestingly, while the full peak width roughly returned to normal-hearing values after 5–7 weeks of deafness, the first half plateaued at approximately 88 % of normal-hearing (one-sample *t* test: *t*_(6)_ = -3.7; *P* = 0.010) and the second half at approximately 105 % hearing (one-sample *t* test: *t*_(6)_ = 2.4; *P* = 0.056). This means that after deafening the eCAP became increasingly skewed.

In Fig. 10D, the normalized peak width and peak area over time are compared to each other for all available eCAPs (recall that peak widths of only 107/150 eCAPs are shown in Fig. 10C). During the first week after deafening, the two measures follow each other remarkably well, but whereas the peak width appears to recover eventually, the peak area logically decreases substantially, analogous to the maximum eCAP amplitude, as the degeneration of SGCs commences (see Table 5).

**Table 5:** rmANOVA results for eCAP peak width and peak area

	**Pre-deafening (*N* = 9)**			**Post-deafening (*N* = 8)**			
	*F*	*df*	*P*	*F*	*df*	*P*	
**Second half peak width**	2.3	3, 24	0.10	9.7	9, 63	< 0.001	
**Second half peak area**	1.1	3, 24	0.37	5.4	9, 63	< 0.001	

## Discussion

In a longitudinal experimental design, we recorded eCAPs in 10 guinea pigs before and after systemic deafening. We could therefore distinguish between transient effects of implantation, acute effects of hair cell loss, and prolonged effects of SGC degeneration on several eCAP measures.

### Absolute eCAP Measures

Compared to their respective IPG effects, the absolute eCAP measures appeared to be more variable over time during the stable period (in terms of cochlear health) before deafening (compare Figs. 6 and 7). Very similar trends after implantation have been observed in eABRs recorded in normal-hearing guinea pigs regarding amplitude (increase), threshold (decrease), and latency (decrease) (Agterberg et al. 2009). Indeed, whereas the absolute eCAP measures may have been influenced by several biological processes triggered by the electrode insertion (e.g., inflammatory reaction to foreign body or insertion trauma [Su et al. 2008; Agterberg et al. 2009]), the IPG effects may have remained stable since they are less affected by non-neural factors (Schvartz-Leyzac et al. 2020b). This, however, does not imply that absolute eCAP measures are unsuitable as an indicator of neural survival. All six eCAP measures shown in Fig. 6 significantly changed after deafening, of which the gradual decrease in maximum amplitude and AGF slope is the most obvious manifestation of ongoing SGC loss. However, the fact that absolute eCAP amplitude, as well as the more indirect measure of AGF slope, consistently reflects SGC survival in animal studies (Hall 1990; Ramekers et al. 2014; Schvartz-Leyzac et al. 2020a; Swiderski et al. 2020; Vink et al. 2020) does not necessarily mean that these measures are useful predictors of SGC survival in human CI users. Substantially more variability is introduced in heterogeneous patient study groups with varying etiology of deafness, extent of hearing loss, age, and type of electrode array, which might significantly reduce the predictive potential of these absolute eCAP measures (e.g., Imsiecke et al. 2021).

### IPG Effects

As discussed in the previous section, before deafening, IPG effects were more stable than the absolute eCAP measures, illustrating their suitability as neural health markers (Fig. 7). After deafening, all measures except for Δthreshold changed significantly, thereby largely confirming our previous findings in acutely implanted normal-hearing (NH) 2-week deaf (2WD) and 6-week deaf (6WD) guinea pigs (Ramekers et al. 2014). Abrupt changes were observed for Δamplitude, Δdynamic range, and Δlevel_50%_ (between day 14 and day 21), while gradual changes over time after deafening (between day 7 and day 28) were observed for Δslope and Δlatency. Specifically, the gradual change in Δlatency (i.e., IPG effect on N_1_ latency) that took place in the first 6 weeks after deafening is highly similar to our previous observations. In that study, mean Δlatency was 6 µs for NH, 14 µs for 2WD, and 24 µs for 6WD animal groups (and 27 µs for 6WD animals in Vink et al. 2020), compared to 5.1 µs, 15 µs, and 26 µs for the animals in the present study for those specific time points, respectively. This gradual change in Δlatency over the course of 4 to 6 weeks after deafening coincides with the loss of ~50 % of the SGC population during the same period of time (Versnel et al. 2007; Agterberg et al. 2008; van Loon et al. 2013; Ramekers et al. 2015a), and may therefore be used as a predictor of neural survival. Alternatively, since Δlatency appears to stabilize beyond 4 weeks after deafening – at least up to 14 weeks after deafening (25 µs in Ramekers et al. 2015a) – it may reflect electrophysiological changes at the individual level as a result of deafness-induced degeneration, such as shrinkage (e.g., Limón et al. 2005), loss of myelin (e.g., Resnick and Rubinstein 2021), or changes in membrane ion channel composition (e.g., Luque et al. 2021). Note that although often suggested as a characteristic phase in the degeneration process of SGCs, in our lab, we do not observe retrograde degeneration, being loss of peripheral processes prior to loss of the SGC soma (Ramekers et al. 2020). As we have argued previously, the magnitude of the latency difference between the two IPG conditions (up to 27 µs) closely resembles the IPG increase itself (27.9 µs), suggesting a preference shift in excitability from first phase of the biphasic current pulse to the second – from normal-hearing to deaf (Ramekers et al. 2014). This hypothesis somewhat resembles one often referred to as polarity sensitivity, asserting that healthy SGCs respond to cathodic currents and (retrogradely) degenerating SGCs to anodic currents (Rattay et al. 2001; Undurraga et al. 2010; Joshi et al. 2017; Hughes et al. 2018; Jahn and Arenberg 2019; Brochier et al. 2021), with the crucial difference that in our lab, all guinea pigs (NH and deaf) respond mainly to cathodic currents (unpublished data), and that they do not lose their peripheral processes prior to losing their SGC soma after deafening (Ramekers et al. 2020).

In the section above, based solely on the latency data, we postulate the hypothesis that the cathodic phase in cathodic-leading pulses is the most excitatory in normal-hearing animals, but that after deafening, this preference shifts to the cathodic (i.e., second) phase in the anodic-leading pulse. As a reminder, it should be noted that since alternating polarity was used for artifact reduction, both pulse polarities have been applied, but that the respective responses cannot be individually assessed. The hypothesized shift in excitation from cathodic-first to cathodic-second occurring simultaneously with SGC degeneration is supported by a smaller IPG effect on both AGF slope and level_50%_ – essentially a smaller enhancement of excitation efficacy. If indeed after deafening the second phase of the biphasic current pulse gradually becomes the predominant excitatory one, the influence of the IPG in separating the two phases becomes increasingly irrelevant, since the IPG then no longer delays the onset of the repolarizing anodic phase – abolishing the preceding action potential generation – but merely delays the excitation itself. The IPG effects on maximum amplitude and dynamic range both increase after deafening, which seemingly contradicts this hypothesis. However, these two effects take place near the upper asymptote of the sigmoidal AGF, and therefore arguably do not reflect the overall excitability of the majority of the SGC population. Rather, a larger maximum amplitude brought about by high stimulation levels may reflect increased neural excitation by the less-effective cathodic-first pulse in the deaf animals.

Although all 10 animals were deaf for exactly 7 weeks, SGC survival did vary across animals. Importantly, even in this small homogenous group of guinea pigs did we find trends in the correlations between IPG effects and SGC survival that were largely similar to those observed previously (Ramekers et al. 2014). Because of the homogeneity of this group, we here for the first time unambiguously show that it is specifically the extent of SGC degeneration, and not for instance the associated duration of deafness, that drives the IPG effects. This is relevant for CI research since although it has been demonstrated repeatedly in histopathological studies (e.g., Spoendlin 1975; Nadol 1990; Nadol et al. 2001; Fayad and Linthicum 2006) that deafness is virtually invariably accompanied by SGC degeneration, the extent of this degeneration can vary substantially.

### Electrode Impedances

Although electrode impedances varied substantially across animals (range ~3.5–6 kΩ at day 0; ~3.5–8 kΩ at day 49), neither absolute eCAP measures nor IPG effects appeared to be influenced by them, which confirms observations by Schvartz-Leyzac et al. (2020a) and Swiderski et al. (2020). Probably, since the stimulation was current-controlled, higher impedances required a larger voltage difference, which is not problematic as long as the limits of the stimulator are taken into account. A possible side effect of higher voltages due to higher electrode impedances for CI users might therefore be larger spread of excitation at similar loudness levels. However, recent studies in CI users have concluded that electrode impedances do not (negatively) influence speech perception scores (Scheperle 2017; Prenzler et al. 2020).

### Enhanced Neural Excitability After Deafening

A series of findings consistent with enhanced neural excitability was observed in the first week after deafening across several (mutually related or unrelated) eCAP measures: a temporarily steeper AGF slope, lower threshold and level_50%_ (which can be interpreted as a populational threshold measure), shorter latency, and narrower N_1_ peak imply that SGCs are excited faster, more easily, and more synchronously several days after deafening than before. Probably, the immediate (functional) loss of hair cells (Versnel et al. 2007) results in abolition of spontaneous neural activity (up to 100–150 Hz; Liberman 1978; Versnel et al. 1990), thereby increasing the susceptibility of the SGC population to extracellular stimulation and consequently their excitability and synchronicity. The less spontaneous activity, the fewer cells are refractory at any given moment, so that more cells are available to be recruited synchronously by a peri-threshold-level stimulus. This effectively lowers the eCAP detection threshold, even though the excitation threshold of individual cells may be elevated by the loss of spontaneous activity (Manley and Robertson 1976).

The observed increased excitability of the auditory nerve furthermore implies that for a full week, the auditory nerve itself remained healthy and thus cannot have been directly targeted by the systemic kanamycin treatment. Rather, the first signs of functional degeneration were observed during the second week after deafening (shallower AGF slope, longer latency, smaller peak area), which coincided with the onset of SGC death previously observed using histological quantification of SGC survival after kanamycin deafening (van Loon et al. 2013). Such delayed neuronal degeneration arguably did not result from direct kanamycin toxicity, but rather resulted from loss of neurotrophic support from the directly targeted organ of Corti (Ramekers et al. 2012).

### Peak Width and Peak Area Represent Different Aspects of SGC Degeneration

The course of the eCAP width over time after deafening (Fig. 10C) resembled that of the N_1_ latency (Fig. 6F) remarkably well. As discussed in the previous subsection, the initial decrease may have resulted from increased synchronicity caused by hair cell loss; the subsequent increase – both wider peak and longer latency – may reflect the presence of a relatively large portion of the SGC population that is still functionally contributing to the eCAP, but is in the process of degeneration. These degenerating SGCs are likely to respond slower to electrical stimulation (e.g., due to demyelination or deteriorated cellular energy homeostasis), and will therefore cause the eCAP to widen, thereby increasing its latency. Finally, a narrowing of the peak accompanied by shorter peak latency then suggests that, once these degenerating cells have died, the surviving SGCs at this point in time are more homogenous once again – a more synchronized and, on average, a healthier population. In other words, peak width and latency may be used to estimate the degeneration rate. This notion of peak width and latency being subject to the relative presence of different subpopulations (in our case healthy and degenerating neurons) has been previously described extensively by Harris et al. (2018) in the context of contributions of two distinct populations of auditory nerve fibers (in their case the high-threshold low-spontaneous rate fibers and low-threshold high-spontaneous rate fibers) in the acoustically evoked CAP.

The peak area, on the other hand, decreased largely monotonically after deafening (Fig. 10D). It therefore resembles the course of the eCAP maximum amplitude and AGF slope (Figs. 6A, B), and is consistent with a reduction in the size of the SGC population.

In summary, the present data suggest that longitudinal changes in the peak width and latency reflect the rate of SGC degeneration, while peak area, maximum amplitude, and AGF slope reflect population size.

### Clinical Implications

Most of the eCAP characteristics had stabilized within 4–6 weeks after deafening, when SGC survival is approximately 50 %, beyond which differences among animals – or within animals over time – would be small. In clinical settings, patients are obviously not typically implanted within mere weeks after onset of hearing loss. We nonetheless consider the present findings to be encouraging for the use of eCAP-based cochlear health measures and estimates of CI outcomes (Schvartz-Leyzac and Pfingst 2018) in clinical practice. The time course of SGC degeneration in humans is much slower than in guinea pigs (e.g., Spoendlin 1975; Nadol 1990), and the correlations in Fig. 8 strongly suggest that the differences in eCAP measures are a function of SGC survival rather than duration of deafness. As argued above, in particular for SGC survival above 50 %, the various eCAP measures may reflect distinct phases in the degeneration process, which may be valuable for clinical diagnostics.

In more general terms, it should be noted that translation of the present data to human CI users may be complicated by obvious anatomical differences between the guinea pig and human cochlea (e.g., cochlear dimensions, myelination of the SGC soma), and that the presently used ototoxicity model may not extrapolate directly to other causes of deafness. In addition, in contrast to human CI users, the animals in the present study were implanted prior to deafening, and did not receive any substantial electrical stimulation (other than for eCAP recordings).

## Abbreviations

ABR: Auditory brainstem response
AGF: Amplitude growth function
CI: Cochlear implant
eCAP: Electrically evoked compound action potential
IPG: Inter-phase gap
NH: Normal hearing
SGC: Spiral ganglion cell
